# A Soft Robotics‐Based Approach to Elbow Rehabilitation: Design, Development, and Validation

**DOI:** 10.1155/abb/6212800

**Published:** 2026-10-17

**Authors:** Janet López-Romero, Alexandro López-González, Juan C. Tejada

**Affiliations:** ^1^ Departament of Engineering Studies for Innovation Universidad Iberoamericana Ciudad de México Mexico ibero.mx; ^2^ Artificial Intelligence and Robotics Research Group (IAR) Universidad EIA Envigado Colombia eia.edu.co

**Keywords:** biomechanics, elbow, pouch motor, rehabilitation, soft robots

## Abstract

This paper presents the design, development, and validation of an innovative soft robotics‐based elbow rehabilitation device. Unlike conventional rigid passive rehabilitators, the proposed system prioritizes safety in human–robot interaction while introducing a novel approach to enhancing elbow mobility. Functional tests were systematically conducted on pouch motors to assess their suitability for integration into the rehabilitator. The device achieved an elbow range of motion of 0°–90° and generated an optimal force of ~98 N at 2 bar of pressure. The experimental results revealed a distinct nonlinear performance behavior, which was thoroughly analyzed to refine system operation and modeling. The study establishes a comprehensive framework encompassing actuator manufacturing, experimental testing, dynamic system identification, and open‐loop control validation. Collectively, these stages culminate in the validation of the proposed dynamic model. Overall, this work represents a meaningful advancement in rehabilitation technology, providing a safer and more adaptable alternative for elbow rehabilitation, with strong potential for application across diverse healthcare settings.

## 1. Introduction

The upper limbs, commonly referred to as the arms, include the shoulder, elbow, wrist, hand, and fingers, enabling a wide range of activities such as social interaction, occupational tasks, recreation, and activities of daily living. Injuries to the upper limbs may significantly impair an individual’s ability to perform these functions depending on their severity. The elbow joint comprises bones, ligaments, tendons, cartilage, muscles, nerves, and bursae, making it particularly susceptible to pain arising from multiple etiologies; injury occurs when any of these structures is compromised [1–3]. Among these conditions, epicondylitis is the most prevalent. This chronic tendinosis results from overuse of the forearm tendons and muscles, producing pain on the lateral side of the elbow that frequently radiates to the forearm. The condition is often accompanied by reduced grip strength, which can lead to substantial functional disability [4, 5].

In addition to epicondylitis, other elbow pathologies, including post‐fracture stiffness, ligament injuries, and postsurgical immobilization, frequently require controlled passive mobilization to prevent joint contractures and long‐term functional deterioration. Early and properly dosed mobilization is clinically recognized as essential for restoring the range of motion, reducing pain, and minimizing the risk of chronic disability. However, ensuring consistent, safe, and comfortable passive therapy outside specialized clinical environments remains a significant challenge, particularly in regions with limited access to rehabilitation services.

In developing countries such as Mexico, rehabilitation adherence presents an additional challenge. Patients frequently discontinue therapy sessions due to time constraints, financial limitations, or discomfort associated with rehabilitation devices. This lack of adherence often results in the loss of therapeutic progress and reduced recovery outcomes [6]. Conventional upper‐limb rehabilitation typically relies on rigid commercial devices designed to assist passive movements. Nevertheless, this approach introduces further concerns as device malfunction—whether due to insufficient maintenance or improper use—may become counterproductive and potentially compromise patient safety [1, 7, 8].

Most currently available elbow rehabilitation devices are based on rigid mechanical architectures or motor‐driven orthoses that employ metallic linkages, hinges, or bulky structural frames to guide the motion. Although these systems can be effective in controlled clinical settings, they often lack adaptability to individual anatomical variability and may generate localized pressure points at the human–device interface. Prolonged use can, therefore, result in discomfort, reduced compliance, and limited suitability for home‐based rehabilitation. Furthermore, many commercially available solutions are costly and mechanically complex, requiring professional supervision and regular maintenance. From a safety perspective, rigid actuation mechanisms may transmit excessive forces in cases of improper adjustment or unexpected patient movement, increasing the risk of secondary injury.

Soft robots are systems composed of compliant materials with a low Young’s modulus (10^2^–10^6^ Pa), enabling them to conform to environmental and anatomical constraints [9]. Their inherent compliance and multiple degrees of freedom allow them to adopt a virtually infinite variety of shapes. Due to their low resistance to compressive forces, soft robots respond more safely to collisions compared with rigid robotic systems [10]. The field of soft robotics draws inspiration from biological organisms, emulating species that have adapted their morphology for survival and replicating the human body’s integration of soft, elastic, and rigid structures within a unified system [9, 11–15]. Based on this biological inspiration, classification systems have been proposed according to animal morphology, distinguishing between structural support and actuator type [16].

The application of soft robots in rehabilitation has grown considerably, particularly in prosthetic and orthotic systems, owing to their flexibility, comfort, safety, and adaptability. These characteristics make them especially suitable for integration into rehabilitation protocols, providing improved human–robot interaction for patients with limited mobility or a restricted range of motion [17–19]. Building upon these advantages, the present design aims to enhance patient comfort during rehabilitation while reducing the risk of device‐related complications. Compared with conventional rigid robotic systems, soft robotic solutions offer superior adaptability to the user’s anatomy and surrounding environment while maintaining equal or greater therapeutic functionality [20–23].

Despite these advances, a significant gap remains in the development of dedicated soft robotic systems for elbow rehabilitation. Many reported devices focus primarily on the hand or wrist, and several existing elbow‐oriented prototypes incorporate complex control architectures or multiple actuator configurations that increase the system cost and mechanical complexity. There remains a need for a rehabilitation device that combines soft actuation with mechanical simplicity, reduced production cost, ergonomic adaptability, and intrinsic safety—particularly for implementation in low‐resource clinical environments and home‐based therapy settings.

In this context, we present a novel passive soft robotic device for elbow rehabilitation that integrates pneumatic pouch motor actuators [24–28]. These actuators are arranged in an array configuration that expands sequentially through controlled air influx, thereby generating smooth and guided motion [29–33]. Ergonomics constituted a central design parameter, ensuring that the device comfortably adapts to different body morphologies through the incorporation of anthropometric measurements [34–38]. Because anthropometric parameters vary according to body composition, sex, age, and ethnic origin, they must be defined locally to ensure appropriate fitting [39–43]. Accordingly, this work considers anthropometric data from the male Mexican population aged 18–64 years residing in Mexico City to guarantee adequate anatomical compatibility [44].

Recent literature has shown an increasing interest in soft robotic rehabilitation systems based on compliant pneumatic actuation. Reviews on soft rehabilitation gloves have highlighted the importance of exploring alternative actuation techniques to improve comfort, safety, and adaptability in upper‐limb assistance. In parallel, soft pneumatic muscle‐based devices have been proposed for post‐stroke rehabilitation, demonstrating the potential of compliant actuators to generate controlled therapeutic movements while reducing the risks associated with rigid mechanisms. Moreover, recent studies have introduced computational optimization approaches, including finite element analysis and neural network‐based methods, to improve the design and performance of soft actuators for rehabilitation applications. In this context, the proposed device contributes to this research direction by integrating a mechanically simple pouch motor array with a flexible splint and a static support structure to assist passive elbow flexion–extension within a targeted rehabilitation range [45–47].

The elbow primarily performs flexion–extension, with a physiological range extending from 0° in full extension to ~145° in maximum flexion. For rehabilitation purposes, however, passive mobilization is often restricted to safer and functionally relevant angular intervals depending on the patient’s condition. In this study, the proposed soft robotic device is designed to assist passive elbow motion within a targeted range of 0°–90°. As shown in Figure 1, the concept consists of a flexible splint coupled to the forearm, a posterior pouch motor array located beneath the forearm to induce flexion, and a static arm support rigidly attached to the base to provide stability during therapy.

**Figure 1:**
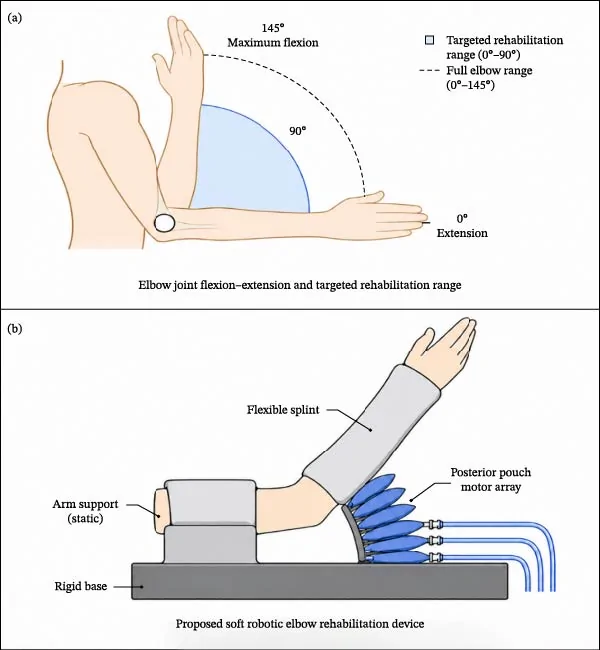
(a) Elbow joint flexion–extension biomechanics, showing the full physiological range of motion (0°–145°) and the targeted rehabilitation range addressed in this study (0°–90°). (b) Conceptual schematic of the proposed soft robotic elbow rehabilitation device, including a flexible splint attached to the forearm, a posterior pouch motor array positioned beneath the forearm to generate passive flexion, a static arm support, and a rigid base.

The main contributions of this work can be summarized as follows:

i. The design and development of a novel passive soft robotic device for elbow rehabilitation based on an array of pneumatic pouch motor actuators, emphasizing ergonomic adaptation through locally defined anthropometric parameters.
ii. The experimental characterization of the pneumatic actuator array to evaluate its expansion behavior, force generation, and capability to produce controlled elbow motion.
iii. The system identification and dynamic analysis of the proposed device under pneumatic actuation to understand its motion response and performance characteristics.
iv. The experimental validation through human testing to demonstrate safe, comfortable, and functional passive elbow mobilization.

These contributions collectively address key limitations of conventional rigid rehabilitation devices by prioritizing compliant actuation, mechanical simplicity, ergonomic fit, and potential cost effectiveness for low‐resource and home‐based rehabilitation environments.

## 2. Methodology

### 2.1. Pouch Motors Actuators Design and Manufacture

Pouch motors are soft fluidic actuators designed for scalable manufacturing and were originally conceived to be fabricated from sheet materials using readily available tools. Structurally, they consist of one or more sealed chambers filled with fluid [48]. These actuators can be manufactured from thermoplastic sheets by stacking two layers and bonding them along their edges through controlled heat application, thereby forming a pouch‐like structure. Two automated fabrication methods are commonly employed to produce different pouch motor geometries: thermal stamping and thermal stretching. Thermal stamping involves a more time‐consuming process as it requires the fabrication and integration of a sealing element into a thermal printing system. In contrast, thermal stretching offers greater flexibility since the actuator geometry can be directly outlined using a thermal marker, thereby simplifying the manufacturing process [49].

In this study, three different pouch motor configurations were evaluated: one fabricated from 1.5 ± 0.05 mm polyethylene fabric, another from 2.5 ± 0.05 mm nylon coated with PVC, and a third from 5 ± 0.05 mm PVC tarp fabric. Tension and compression tests using fixed weights were conducted to assess their mechanical performance. The initial results indicated that fabric‐based actuators (nylon and PVC) outperformed those fabricated from polyethylene. In particular, nylon demonstrated superior suitability for heat sealing, as illustrated in Figure 2a, consistent with findings reported in the pouch motor literature [49]. However, subsequent testing revealed that the heat‐sealed nylon joints exhibited structural weakness, failing to withstand internal pressures approaching 1 ± 0.05 bar and showing a tendency to rupture under the applied loading conditions.

**Figure 2:**
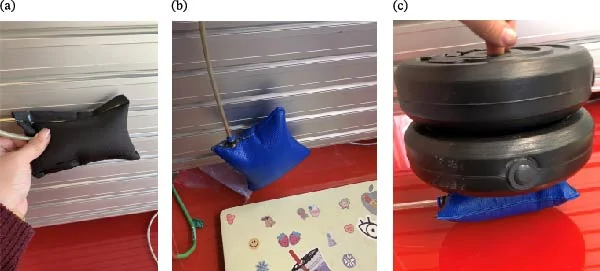
Pouch motors fabricated from different fabrics. (a) PVC pouch motor. (b) Pouch motor PVC fabric. (c) Loading test.

To ensure reproducibility of the actuator evaluation, all tests were performed using the same geometric configuration (15 ± 0.5 cm × 15 ± 0.5 cm actuator footprint) and under controlled inflation and loading procedures. Each actuator was connected to a pneumatic line through a standard inflation valve, and internal pressure was monitored throughout the tests using an inline pressure gauge mounted between the air source and the actuator. Pressure was increased in small increments and held constant during each measurement step to allow the actuator to reach a steady‐state deformation before recording the data. Displacement was quantified as the change in actuator thickness (out‐of‐plane expansion) under load, measured with a digital caliper at the actuator centerline, and additionally verified by side‐view image capture using a fixed camera position and a calibrated ruler placed in the same plane.

The tensile and compressive responses of each actuator were evaluated under incremental loading conditions using calibrated fixed weights, as shown in Figure 2c. To ensure repeatability and reproducibility of the results, each test was conducted over multiple loading–unloading cycles. This procedure allowed for the assessment of consistency in actuator behavior under repeated mechanical stress. The detailed results of these measurements, including the total number of cycles performed for each actuator configuration, are presented in Section 3.

For each load condition, the measurement sequence consisted of (i) setting the target pressure, (ii) applying the corresponding fixed weight, (iii) recording the stabilized displacement after a short settling period, and (iv) fully deflating the actuator before starting the next repetition. This inflate–load–measure–deflate sequence was repeated for multiple cycles to capture repeatability and potential hysteresis effects. In addition to the steady‐state displacement, qualitative observations such as seam behavior, material wrinkling, and localized overstress regions were documented.

Subsequent tests were conducted using a PVC tarp, as shown in Figure 2b, a material widely used in the manufacture of inflatable structures. This fabric was selected due to its polyester base coated with PVC, which provides high resistance to environmental variations and mechanical stress. Its dense weave and substantial thickness allowed the use of sewing as an alternative to heat sealing as the tight thread structure ensured that stitching did not compromise structural integrity or significantly affect air retention [49]. The PVC‐based pouch motors were fabricated by cutting two identical square sheets of material, which were then stitched together using industrial sewing machines. A straight stitch with reinforced corners was applied, leaving a small unstitched section to allow the assembly to be turned inside out, thereby concealing the seams and improving structural robustness. After inversion, the entire perimeter was sewn again using a straight stitch, leaving a designated opening for the inflation valve. The valve was subsequently secured to the fabric using an epoxy adhesive to ensure airtight sealing. Each pouch actuator had nominal dimensions of 150 ± 0.05 mm in length, 150 ± 0.05 mm in width, and 5 ± 0.05 mm in thickness, ensuring geometric consistency across samples and supporting reproducibility in the experimental evaluations.

Failure criteria were defined a priori to standardize the comparison across materials. Specifically, actuator failure was declared when any of the following occurred: (i) visible rupture or bursting of the pouch body; (ii) seam separation or tearing that produced a sudden pressure drop; (iii) persistent air leakage evidenced by an inability to maintain the target pressure during the holding period; or (iv) irreversible deformation or material fatigue (e.g., progressive seam elongation or delamination) that resulted in a measurable loss of repeatability across cycles at the same pressure and load. For safety, pressure increments were halted immediately upon detection of any failure indicator.

To determine the load‐bearing capacity of the actuator, incremental weights were progressively applied until observable signs of material or seam fatigue appeared. The experimental results confirmed that the actuator was capable of withstanding a load of 4 ± 0.1 kg at an internal pressure of 2 ± 0.01 bar without exhibiting structural deformation, seam separation, or rupture. Based on these findings, an array configuration of pouch motors was developed to operate in a rotational arrangement. The actuators were stacked in series so that they exerted force against one another, thereby increasing the overall load‐supporting capability of the system. This actuator array was subsequently integrated into an elbow brace structure, as illustrated in Figure 3a. Following multiple configuration trials, the arrangement positioning the pouch motors along the posterior region of the arm was selected over the inner arm configuration shown in Figure 3a. This placement enabled greater elbow flexion with reduced actuation effort, as demonstrated in Figure 3b, improving the mechanical efficiency and functional performance of the device.

**Figure 3:**
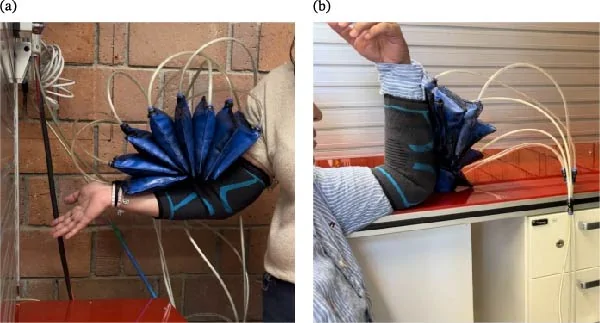
Tests of the array of pouch motors. (a) In elbow brace. (b) On the back of the arm.

### 2.2. Soft Splint Design

The splint design was based on anthropometric data of the Mexican male population aged 18–64 years [44], which provided quantitative reference values for upper‐limb segment dimensions, including arm length, forearm length, elbow height, and proportional relationships between anatomical landmarks. These anthropometric parameters were used to define the geometric proportions of the splint, ensuring anatomical correspondence between the upper arm and forearm sections and establishing a biomechanically coherent alignment with the elbow joint axis. By grounding the design on population‐specific anthropometric data, dimensional accuracy and ergonomic compatibility were systematically incorporated into the development process.

Although the system was intended for use on a fixed surface, the attachment and assembly method were kept straightforward to facilitate reproducibility and ease of use. To guide the design process, sketches were first created to visualize the appearance and structural configuration of the splint. An initial idea considered the use of pneumatic cushions to enhance hand and arm support; however, this concept was later replaced by covering the entire splint with memory foam cushions. This modification improved comfort, provided a more uniform pressure distribution, allowed better adaptation to different arm morphologies, and reduced manufacturing costs, as presented in Figures 4 and 5.

**Figure 4:**
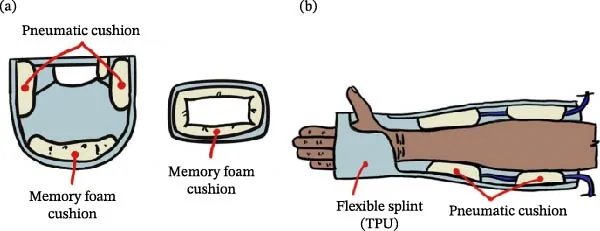
Sketch of the soft splint and its components. (a) Front view of the splint. (b) Top view of the splint.

**Figure 5:**
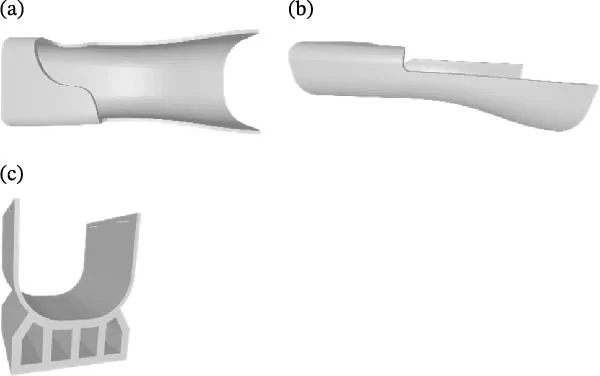
Design of the soft splint and its components. (a) Side view of the splint. (b) Top view of the splint. (c) Arm support.

As illustrated in Figure 6, the final 3D design of the soft splint includes an additional arm support that was also 3D‐printed and secured to a stable, fixed base to prevent unwanted movement. Both the splint and the support were manufactured using thermoplastic polyurethane (TPU), a material selected for its flexibility and adaptability. Compared to rigid alternatives such as polylactic acid (PLA), TPU provides controlled compliance, which is essential in soft robotic orthotic systems to accommodate small misalignments between the anatomical elbow axis and the mechanical structure.

**Figure 6:**
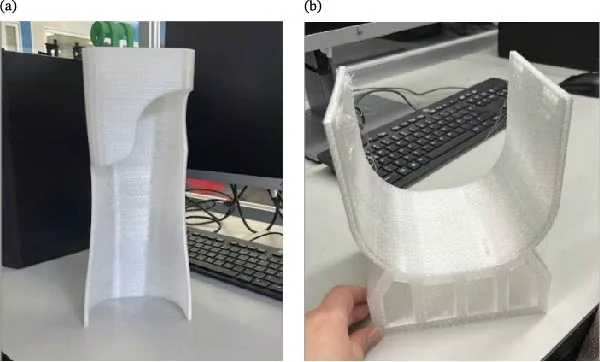
Design of the soft splint and its components. (a) Soft splint printed in TPU. (b) Soft arm support printed in TPU.

Regarding actuator integration, the pneumatic soft actuator was positioned longitudinally along the forearm segment and aligned with the sagittal plane to assist elbow flexion–extension. The elbow joint was defined as the primary axis of rotation during biomechanical analysis. The actuator’s line of action was established considering the perpendicular distance (lever arm) between the force vector and the elbow center of rotation, ensuring effective torque generation while minimizing undesired rotational components. The combination of anthropometric alignment, compliant materials, and controlled actuator positioning reduces mechanical mismatch and improves the overall ergonomic performance.

### 2.3. Integrated Design

For the integration of the soft splint with the pouch motor array, the actuators were secured to the posterior surface of the splint using high‐strength, industrial‐grade, outdoor‐rated Velcro to ensure reliable attachment under repeated loading conditions. The opposite end of each actuator was likewise fixed using Velcro to a rigid wooden board, providing a stable and immovable anchoring surface to prevent undesired displacement during actuation. To improve user comfort and reduce localized pressure on the arm, memory foam padding was installed along the inner surface of the splint and secured with Velcro, as shown in Figure 7. This cushioning layer enhanced the ergonomic adaptation and improved contact distribution between the device and the user’s limb. Additionally, a McKibben‐type pneumatic artificial muscle fabricated from latex tubing was incorporated as an auxiliary adjustment mechanism to enhance splint fixation around the arm, as illustrated in Figure 8. This element provided adaptable compression, allowing better conformity to different arm geometries. A 3D‐printed circular air distribution manifold with a single inlet and multiple outlet ports was connected to the actuators, as depicted in Figure 9. This configuration enabled a single servo‐controlled valve to simultaneously supply pressurized air to all pouch motors and the McKibben muscle, ensuring synchronized pneumatic actuation across the system.

**Figure 7:**
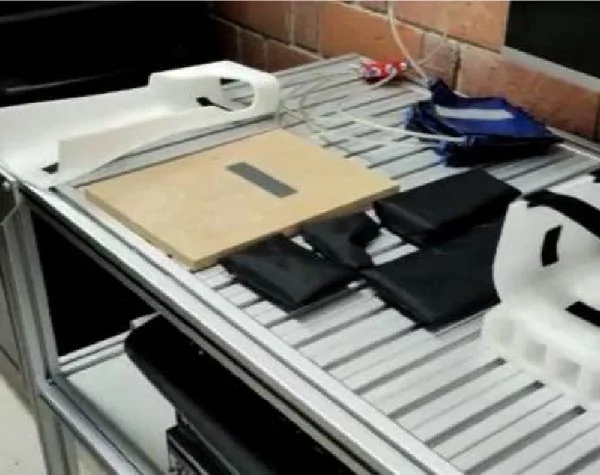
Modulated elbow rehabilitator.

**Figure 8:**
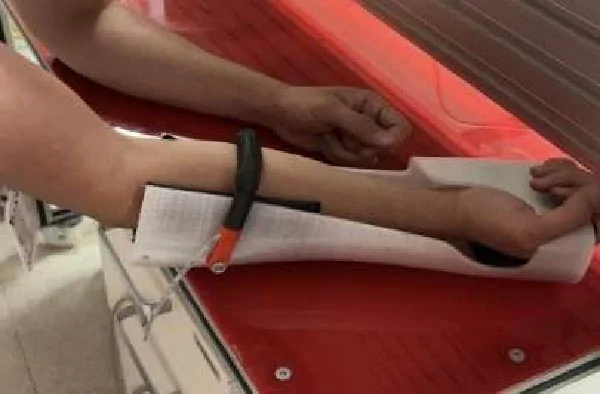
McKibben muscle as an adjustment system.

**Figure 9:**
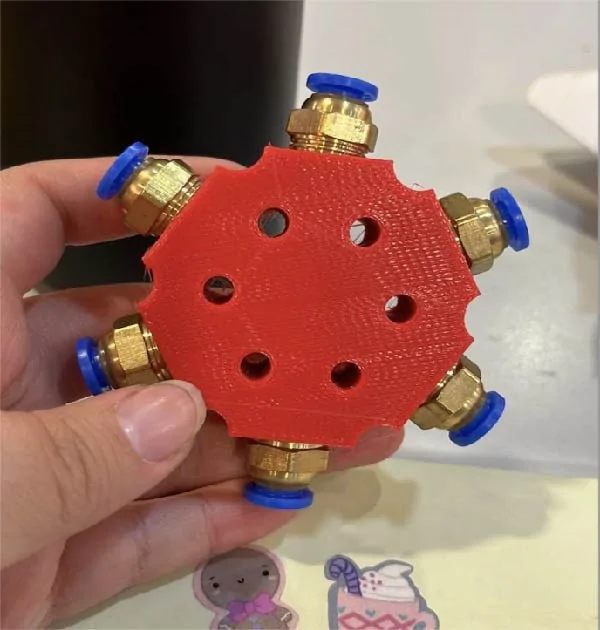
3D‐printed circular air distribution block.

The integration of the mechanical and electronic subsystems was achieved using a 3D‐printed needle‐type servo valve actuated by a stepper motor. This configuration was selected because a needle valve provides finer control resolution compared to conventional valves as it allows a greater number of incremental turns, enabling more precise regulation of small air flow rates. The stepper motor controlled the degree of valve opening, and a calibrated relationship was established between the percentage of valve opening and the motor’s angular displacement. This relationship was implemented and managed through an ESP32 microcontroller, which coordinated the actuation commands and system operation. The final experimental prototype consisted of the rehabilitation device, the servo‐controlled flow regulation valve, a structural support frame, and the pneumatic supply system, as illustrated in Figure 10. A demonstration of the integrated prototype and its operation is provided in Supporting Video S1.

**Figure 10:**
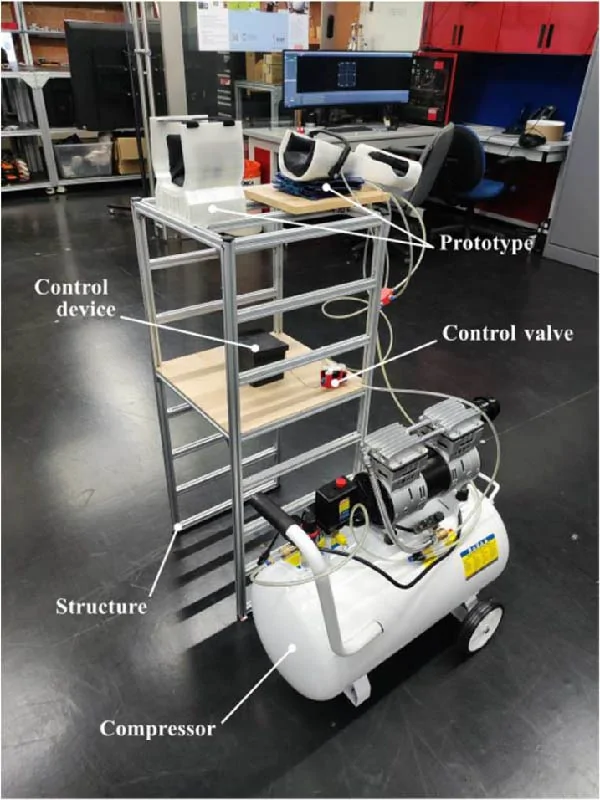
Final testing prototype.

Figure 11 illustrates the integration of the pneumatic and electronic subsystems controlled by the ESP32 microcontroller. The air compressor supplies pressurized air to a distribution manifold (red), which directs the airflow toward the array of pouch motors (blue). The airflow was regulated by a servo‐controlled needle valve actuated through a stepper motor driver. The ESP32 microcontroller generates control signals to the motor driver, which adjusts the angular position of the stepper motor to modulate the valve opening percentage. This modulation directly regulates the airflow supplied to the actuators and, consequently, the internal pressure within the pouch motors. This configuration enables precise and repeatable pressure control, ensuring consistent flexion–extension movements of the rehabilitator during experimental operation.

**Figure 11:**
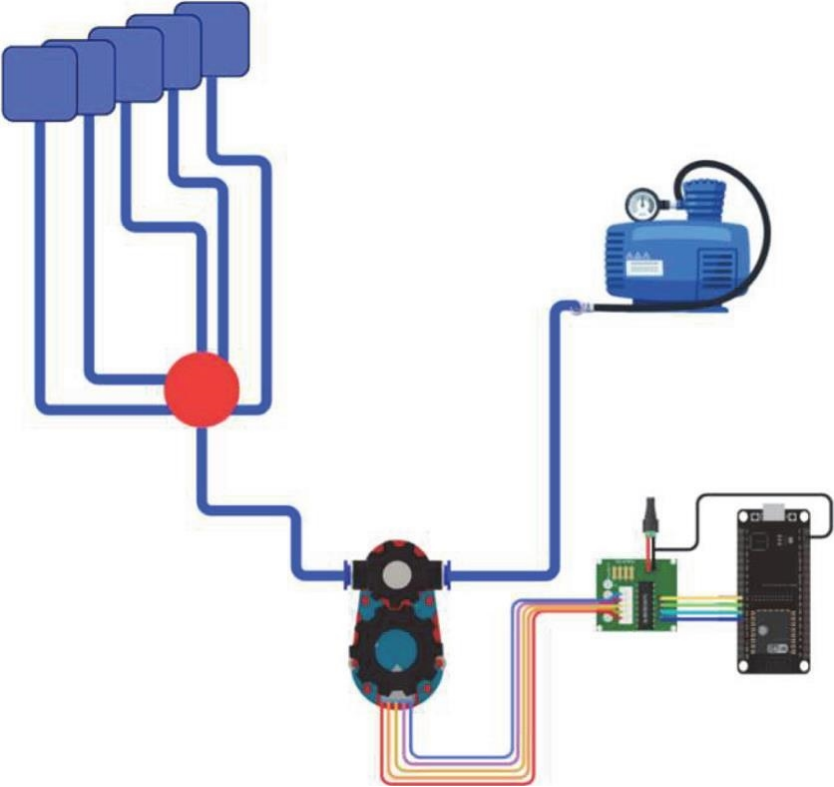
Functional diagram of the rehabilitator system.

Table 1 summarizes the electronic and pneumatic components used in the system, including their respective models and key operational parameters to ensure reproducibility. The pneumatic supply is provided by a 1 HP air compressor (127 V/60 Hz) with a maximum pressure of 116 PSI and a flow rate of 160 LPM (5.65 CFM), which guarantees sufficient airflow capacity for the simultaneous actuation of the pouch motor array. Air distribution is achieved through 6 mm pneumatic tubing and connectors rated up to 1 MPa, ensuring safe operation within the working pressure range of the actuators.

**Table 1:** Electronic and pneumatic components with corresponding models and key operational specifications.

Component	Model/type	Key specifications
Air compressor	Marietta	Voltage: 127 V/60 Hz Power: 1 HP Flow rate: 160 LPM (5.65 CFM) Maximum pressure: 116 PSI
Pneumatic connector	6 mm quick connector	Material: Plastic resin Internal body: Stainless steel Diameter: 6 mm Max. pressure: 1 MPa Max. Negative pressure: –750 mmHg Operating temperature: 0–60°C
Pneumatic tubing	—	Diameter: 6 mm Length: 50 cm
Servo valve (custom‐built)	3D printed (PLA) + Needle gas valve (Cufiat)	Type: Pneumatic *T*‐valve Diameter: 6 mm Rotation range: 360° Actuation force: ≈120 N Max. pressure: ≥1.0 MPa
Stepper motor	28BYJ‐48	Voltage: 5 V Step angle: 5.625° × 1/64 (gear reduction)
Motor driver	ULN2003	Voltage: 5 V Current capacity: 500 mA Digital control pins: 7
Microcontroller	ESP32	Operating voltage: 3.3 V Connectivity: Wi‐Fi, Bluetooth LE, and 2.4 GHz transceiver Digital I/O pins: 14 Analog inputs: 8 Communication protocols: SPI, I2C, I2S, UART, and CAN (TWAI)
AC adapter	—	Input voltage: 100–240 V Output voltage: 5 V Output current: 2 A Port: USB
Fabric	PVC‐coated textile	UV protection Knitted (closed structure) Fire‐retardant properties

Flow regulation is performed using a custom‐built servo valve composed of a 3D‐printed PLA housing integrated with a multi‐turn needle valve (T‐type configuration, 6 mm diameter) capable of operating at pressures ≥1.0 MPa. The needle valve design allows fine adjustment of airflow, which is mechanically actuated by a 28BYJ‐48 stepper motor (5 V, 5.625° × 1/64 step angle). The motor was driven by a ULN2003 driver module (5 V, 500 mA), enabling precise control of angular displacement and, therefore, proportional modulation of valve opening.

The control architecture is based on an ESP32 microcontroller operating at 3.3 V, selected for its enhanced processing capabilities and integrated Wi‐Fi/Bluetooth communication. The ESP32 provides 14 digital I/O pins, 8 analog inputs, and multiple communication protocols (SPI, I2C, I2S, UART, and CAN), allowing flexible integration of sensing and control elements. The microcontroller generates the control signals that determine the stepper motor position and, consequently, the valve opening percentage. Power regulation is ensured through a 5 V/2 A AC adapter supplying both the control electronics and driver circuitry.

By explicitly detailing these models and parameters, the revised manuscript improves technical transparency and enables replication of the pneumatic–electronic architecture described in Figure 11.

### 2.4. Human Subject Testing and Elbow Angle Measurement Protocol

To evaluate the functional performance of the proposed soft robotic elbow rehabilitation device, experimental tests were conducted with human participants under controlled laboratory conditions.

A total of *n* = 5 healthy adult volunteers (three males and two female) were recruited for this study. Inclusion criteria required participants to be between 28 and 32 years old, with no history of musculoskeletal injury, neurological disorder, or pain affecting the upper limbs. Exclusion criteria included prior elbow surgery, current upper‐limb pain, restricted range of motion, or any medical condition contraindicating passive joint mobilization.

The study protocol was reviewed and approved by the *Comité de Ética de la Universidad EIA* (approval number P202105‐05), approval date January 16th, 2023. All participants provided written informed consent before participation. The procedures followed institutional ethical standards and the principles outlined in the Declaration of Helsinki.

During testing, participants were seated in an upright position with the shoulder in a neutral position and the forearm in a relaxed posture. The soft robotic device was secured to the participant’s arm using adjustable straps to ensure consistent alignment with the elbow joint axis. Care was taken to align the actuator array along the posterior aspect of the arm to facilitate the flexion movement.

Reflective markers were placed on anatomical landmarks to compute elbow joint kinematics. Specifically, markers were positioned at the lateral acromion (shoulder reference point), the lateral epicondyle of the humerus (elbow joint center approximation), and the radial styloid process (wrist reference point). Marker trajectories were recorded using a VICON system operating at 120 Hz.

## 3. Results and Discussion

### 3.1. Actuator Characterization

The quantitative characterization of the pouch motor actuators is summarized in Table 2, which reports the material thickness, the maximum compressive and tensile forces recorded during testing, and the number of measurement cycles performed to assess robustness and repeatability. The polyethylene actuator withstood 1 ± 0.1 kg (~9.8 N) in compression and 5 ± 0.1 kg (~49.0 N) in tension for up to 13 cycles at an internal pressure of 1 ± 0.01 bar without failure; however, it exhibited a comparatively lower overall mechanical strength. Although nylon is described in the literature as a suitable material for heat sealing, the nylon‐based actuator demonstrated limited structural integrity in practice, failing after only three unloaded cycles at ~1 bar due to seam weakness. In contrast, the PVC tarp actuator exhibited the highest stability and load‐bearing capacity among the tested materials. It withstood 4 ± 0.01 kg (~39.2 N) in compression and 9 ± 0.1 kg (~88.3 N) in tension for 13 cycles at 2 ± 0.1 bar without structural failure, confirming its superior mechanical performance and suitability for integration into the rehabilitation system.

**Table 2:** Mechanical characterization of pouch motor actuators to 1 bar.

Actuator material	Thickness (mm)	Tension (N)	Compression (N)	Number of cycles tested
Polyethylene fabric	1.5	49.0 ± 0.10	9.8 ± 0.10	13
Nylon with PVC coating	2.5	—	—	3^a^
PVC tarp fabric	5	88.3 ± 0.10	39.2 ± 0.10	13

^a^The nylon actuator failed at pressures close to 1 bar due to weakness in the heat‐sealed joints, which limited the number of completed test cycles.

The mechanical verification of the pouch motor actuators at an internal pressure of 2 ± 0.01 bar is summarized in Table 3, which reports the maximum compressive and tensile loads sustained, their corresponding force equivalents, and the number of measurement cycles completed. The polyethylene actuator withstood 1 ± 0.1 kg (~9.8 N) in compression and 5 ± 0.1 kg (~49.0 N) in tension; however, it failed after only nine cycles under both loading conditions, confirming its limited structural robustness at elevated pressure. The nylon actuator was not subjected to further testing at 2 bar due to its premature failure at ~1 bar, which indicated insufficient structural integrity for higher‐pressure evaluation. In contrast, the PVC tarp actuator demonstrated superior mechanical performance, sustaining 4 ± 0.1 kg (~39.2 N) in compression and 9 ± 0.1 kg (~88.3 N) in tension for 13 cycles without exhibiting structural failure. These results confirm its suitability for operation under higher‐pressure conditions within the rehabilitation system.

**Table 3:** Mechanical characterization of pouch motor actuators to 2 bar.

Actuator material	Thickness (mm)	Tension (N)	Compression (N)	Number of cycles tested
Polyethylene fabric	1.5	49.0 ± 0.10	9.8 ± 0.10	9
PVC tarp fabric	5	88.3 ± 0.10	39.2 ± 0.10	13

Based on these experimental results, the rehabilitator achieved a functional elbow range of motion of 0°–90°, corresponding to the physiological flexion range of a healthy human elbow. Regarding force generation, the PVC tarp actuator exhibited optimal performance, sustaining tensile loads of up to ~98 N at an internal pressure of 2 bar, as reported in Table 2, without structural failure. This combination of a sufficient range of motion and load‐bearing capacity demonstrates that the prototype can reproduce physiologically relevant movements while maintaining structural integrity and operational safety. These findings validate the mechanical suitability of the proposed system for elbow rehabilitation applications.

In addition to the static load tests, the PVC tarp actuator was subjected to progressive loading–unloading protocols to evaluate its stability under varying pressure conditions. In compression, the applied load was gradually increased from 1 to 4 kg, while in tension, it was incrementally increased from 1 to 9 ± 0.1 kg. Once the maximum load was reached, the weight was progressively reduced to complete the cycle. This procedure was systematically repeated at internal pressures of 1, 2, 3, 4, 5, and 6 bar. As shown in Figure 12, the actuator consistently maintained structural integrity throughout these tests, demonstrating not only its ability to withstand elevated loads but also its resilience under repeated loading–unloading cycles across a broad pressure range. For subsequent system characterization, an operating condition of 4 kg at 2 bar was selected as the reference point. This configuration provided adequate load capacity while preventing excessive mechanical stress on the actuator, thereby enhancing repeatability and minimizing the risk of premature structural degradation. These findings further confirm the robustness, durability, and reliability of the PVC tarp as the most suitable material for integration into the rehabilitation prototype.

**Figure 12:**
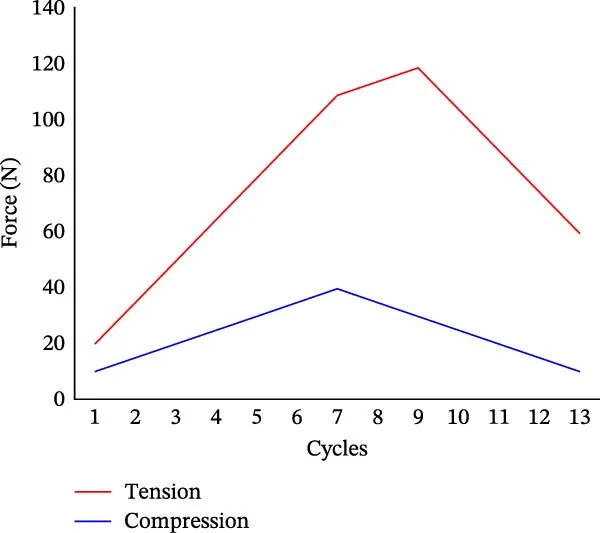
Compression and tension tests of the PVC tarp actuator under progressive loading–unloading cycles.

To verify the functionality of the prototype and conduct its dynamic characterization, the integrated system was assembled and tested at the Motion Analysis Laboratory of Universidad Iberoamericana. The experimental evaluation focused on assessing the system’s achievable range of motion and its dynamic response under different input conditions. Reflective position markers were placed on the device and anatomical reference points to enable motion tracking using the VICON motion capture system. The markers were strategically positioned to ensure the accurate identification of the limb segments and precise reconstruction of elbow joint kinematics. An open‐loop operation was implemented using a control program developed in MATLAB within the Simulink environment. This program acquired the elbow joint angle around its axis of rotation, stored the measurements in a data vector, and saved them for subsequent analyses. Additionally, the program generated and transmitted reference signals to regulate the valve opening. These control inputs ranged from 0% to 100%, corresponding directly to the percentage of valve opening.

The open‐loop control architecture is illustrated in Figure 13.

**Figure 13:**
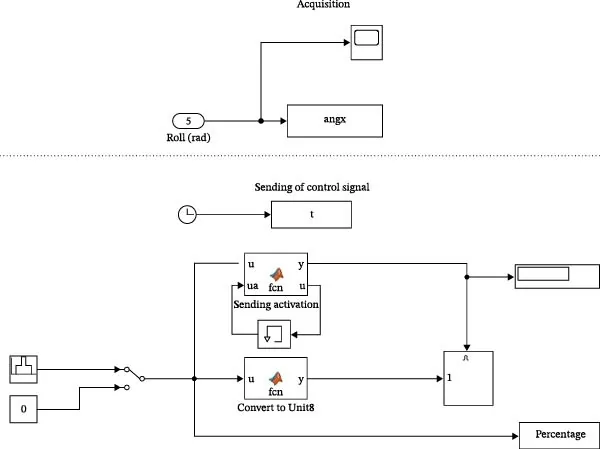
Control and data acquisition framework in MATLAB.

### 3.2. System Stability Verification With Step Inputs

To evaluate the stability of the rehabilitation system, step input tests were conducted using the valve opening sequence of 0%, 50%, 100%, 50%, and 0%. The results of this experiment are shown in Figure 14.

**Figure 14:**
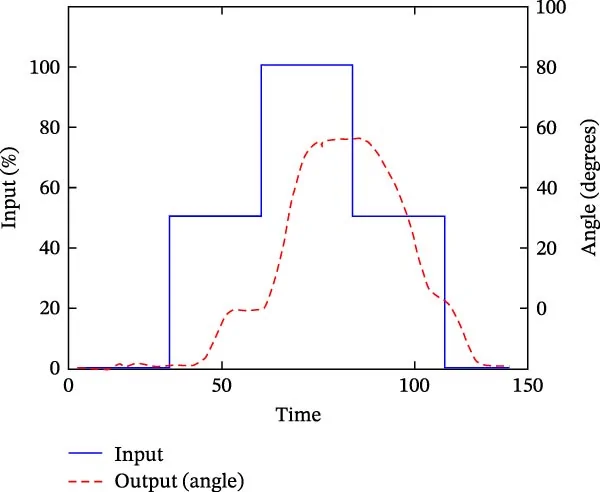
Results of the response test to an input sequence.

The first step corresponds to a 50% valve opening. The input is subsequently increased to 100% opening. Once the maximum angular displacement is reached, the input is reduced to 50% and finally to 0% to conclude the test.

For the system to be considered stable, its response must converge to a steady‐state value without divergence or sustained oscillations. From the experimental observations, both input and output remained finite and bounded throughout the test. According to the bounded‐input bounded‐output (BIBO) stability criterion, the system is therefore considered stable.

Once the stability was verified, system identification was performed.

### 3.3. System Identification

Once the stability of the system was verified, a characterization test was conducted to identify the dynamic behavior of the elbow rehabilitation device. The purpose of this test was to analyze the system’s response to variations in actuator input flow, generated through step‐like signals programed in the control interface.

For the experimental procedure, the subject was properly positioned within the rehabilitation device, and the restraint mechanisms were adjusted to ensure consistent alignment and repeatability. The system was then excited using incremental step inputs of a 10% valve opening. Each input level was maintained until the system reached steady‐state conditions before proceeding to the next increment, thereby ensuring that transient and steady‐state behaviors were adequately captured.

Following the acquisition of the step response data, a mathematical model was identified to represent the system dynamics. The experimental results obtained from the 10% incremental steps are presented in Figure 15.

**Figure 15:**
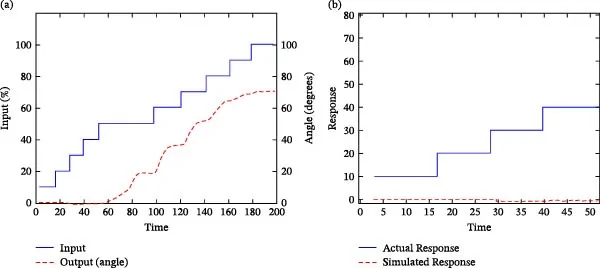
Results of the uphill rehabilitator characterization test. (a) Uphill from 0% to 100% input. (b) Uphill from 0% to 40% zone.

A dedicated analysis of the low‐input region revealed a clear dead zone between 0% and ~40% valve opening, as shown in Figure 15b. Within this range, the system exhibits negligible angular displacement despite increases in the control signal. This behavior indicates that a minimum pressure threshold must be reached before mechanical motion occurs.

Physically, this dead zone can be attributed to several factors. First, a minimum internal pressure is required to overcome the structural stiffness of the pouch actuator material. Second, pneumatic compressibility effects delay the transmission of the effective force. Third, internal friction within the fabric layers and stitching introduce additional resistance. Finally, the gravitational torque of the forearm‐device assembly must be exceeded before measurable elbow rotation can occur. Only after surpassing this combined threshold does the actuator generate sufficient force to produce an observable joint movement. For this reason, system identification and open‐loop modeling were restricted to the 40%–100% valve opening range, where the device exhibits a consistent and measurable dynamic behavior.

For control implementation, it is essential to determine the required valve opening corresponding to the desired rehabilitation angle. To establish this relationship, the steady‐state angular displacement associated with each input step was extracted from the experimental data, as shown in Figure 15 and summarized in Table 4.

**Table 4:** Stationary values at step input.

Angle (°)	Opening real (%)	Opening fit (%)	Fit error (%)
0	40	40.0982	−0.0982
18.7	50	49.3929	0.6071
36.2	60	61.6224	−1.6224
51.5	70	67.6833	2.3167
64	80	81.4282	−1.4282
68.4	90	91.9252	−1.9252
70.3	100	97.8498	2.1502

Using these steady‐state input–output pairs, a fourth‐order polynomial regression was performed to obtain an analytical expression relating valve opening to the rehabilitation angle. The regression was carried out in MATLAB using the polyfit function. The resulting fitted curve is presented in Figure 16, and the corresponding characteristic equation is provided in:

**Figure 16:**
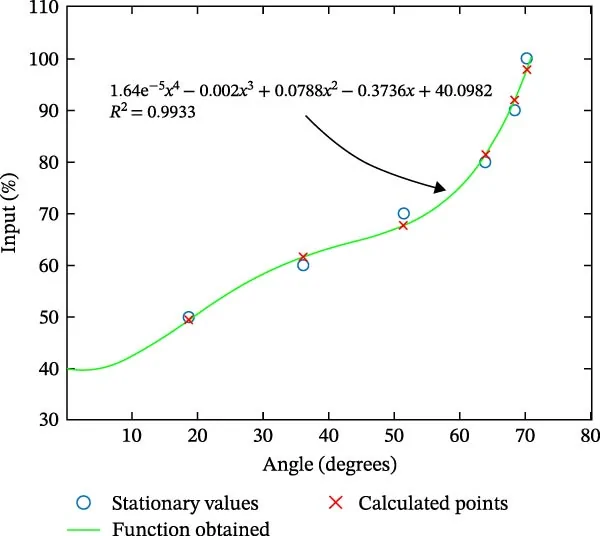
Polynomial regression results.

(1) fx=1.640.0020.07880.373640.0982e−5x4−x3+x2−x+.

The polynomial regression was performed using *n* = 7 steady‐state input–output data points extracted from the experimental characterization. Table 4 presents the real valve opening values, the fitted values obtained from the fourth‐order polynomial, and the corresponding fitting errors.

The maximum absolute fitting error observed was 2.32%, corresponding to the 70% opening case. The mean absolute error (MAE) across all points was ~1.44%, and the root mean square error (RMSE) was ~1.70%. These values indicate that the polynomial model provides an accurate approximation of the steady‐state relationship between valve opening and the rehabilitation angle within the operational range.

### 3.4. First‐Order Approximation as Initial Model

Given the monotonic rise observed in several step responses, the system was initially approximated as a first‐order model. This choice was motivated by the need for a simplified representation capable of capturing dominant dynamics such as static gain and time constant while maintaining analytical tractability.

Each step response was therefore modeled using the following:

CsRs=Kτs+1e−θs,

where *K* is the system gain, *τ* is the system time constant, and *e* ^−*θ* *s*^ is the system delay.

For the identification of parameters for each step, it was determined that

• $K=\frac{\text{Output}}{\text{Input}}$.
• $\tau =\frac{T_{\text{ss}}}{4}$.
• Considering system delays as 0.

The system’s settling time (*T*_ss_) was determined using the 5% stability criterion, meaning the time at which the system reaches 95% of its steady‐state value. Thus, for each step shown in Figure 17, the obtained values and transfer functions are presented in Table 5. To verify the accuracy of the non‐parametric identification, the experimentally obtained responses were compared with the responses of the identified non‐parametric model. The results of this comparison can be observed in Figure 17.

**Figure 17:**
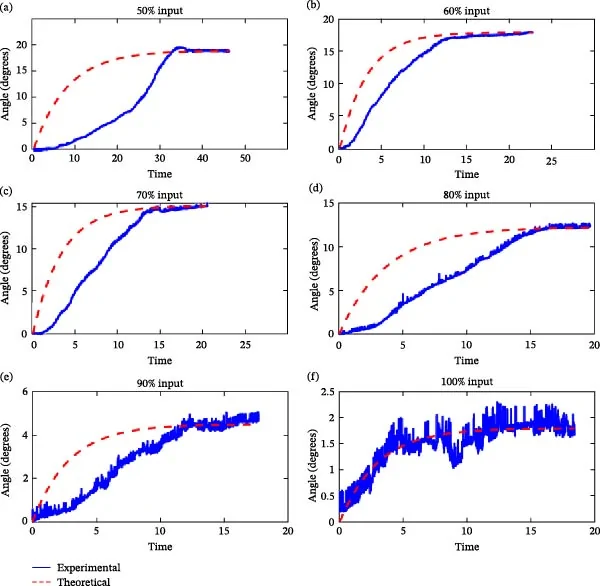
Comparison of experimental and simulated responses of the system with non‐parametric model. (a) Experimental vs. theoretical response at 50% input. (b) Experimental vs. theoretical response at 60% input. (c) Experimental vs. theoretical response at 70% input. (d) Experimental vs. theoretical response at 80% input. (e) Experimental vs. theoretical response at 90% input. (f) Experimental vs. theoretical response at 100% input.

**Table 5:** First order Non‐parametric model for each of the steps.

Step	*K*	*τ*	Transfer function
40–50	1.87	7.95	$\frac{1.87}{7.951s+}$
50–60	1.79	3.20	$\frac{1.79}{3.21s+}$
60–70	1.51	3.39	$\frac{1.51}{3.391s+}$
70–80	1.23	3.71	$\frac{1.23}{3.711s+}$
80–90	0.45	2.88	$\frac{0.45}{2.881s+}$
90–100	0.18	2.99	$\frac{0.18}{2.991s+}$

Although the first‐order model provided reasonable agreement for the 100% step input, it failed to accurately represent responses at intermediate input levels. Specifically,

• Overshoot was observed in certain responses.
• Settling times varied nonlinearly with the input amplitude.
• Gain values changed significantly across the operating regions.
• A dead zone was identified between 0% and 40% valve opening.

These behaviors indicate nonlinear and higher‐order dynamics that cannot be captured by a single dominant pole model.

Therefore, a more representative model was required.

### 3.5. Transition to Second‐Order Modeling

Detailed examination of the experimental responses revealed that

• The 50% step exhibited characteristics of an underdamped second‐order system.
• Steps between 60% and 90% displayed an overdamped behavior.
• The 100% step could still be reasonably approximated by a first‐order response.

To capture these dynamics, the system was modeled using the second‐order transfer function:

CsRs=Kwn2s2+2ζwns+wn2,

where *K* is the static system gain, *w*_*n*_ is the undamped natural frequency, and *ζ* is the damping ratio.

This model allows the representation of overshoot, damping effects, and nonlinear gain variations.

The parameters obtained for the underdamped response are shown in Table 6.

**Table 6:** Non‐parametric model for steps with underdamped second‐order response.

Step	*K*	*T*_ss_	*M*_*p*_	Transfer function
40–50	1.87	31.79	0.0321	$\frac{0.05431}{s^{2}+0.25170.02905s+}$

The non‐parametric identification for overdamped second‐order systems provides the results shown in Table 7.

**Table 7:** Non‐parametric model for steps with overdamped second‐order response.

Step	*K*	*τ*	Transfer function
50–60	1.79	3.20	$\frac{0.733}{s^{2}+1.2550.3722s+}$
60–70	1.51	3.39	$\frac{0.5326}{s^{2}+1.1960.3527s+}$
70–80	1.23	3.71	$\frac{0.371}{s^{2}+1.1190.3017s+}$
80–90	0.45	2.88	$\frac{0.2172}{s^{2}+1.390.4826s+}$

The results of the non‐parametric identification for the different steps of the system are presented in Table 8.

**Table 8:** Second order non‐parametric model for each of the steps.

Step	Transfer function
40–50	$\frac{0.05431}{s^{2}+0.25170.02905s+}$
50–60	$\frac{0.702}{s^{2}+1.2550.3922s+}$
60–70	$\frac{0.5326}{s^{2}+1.1960.3527s+}$
70–80	$\frac{0.371}{s^{2}+1.1190.3017s+}$
80–90	$\frac{0.2172}{s^{2}+1.390.4826s+}$
90–100	$\frac{0.18}{2.991s+}$

The accuracy of the corrected non‐parametric identification can be verified by comparing the experimentally obtained responses with those of the identified non‐parametric model. The results of this comparison are shown in Figure 18.

**Figure 18:**
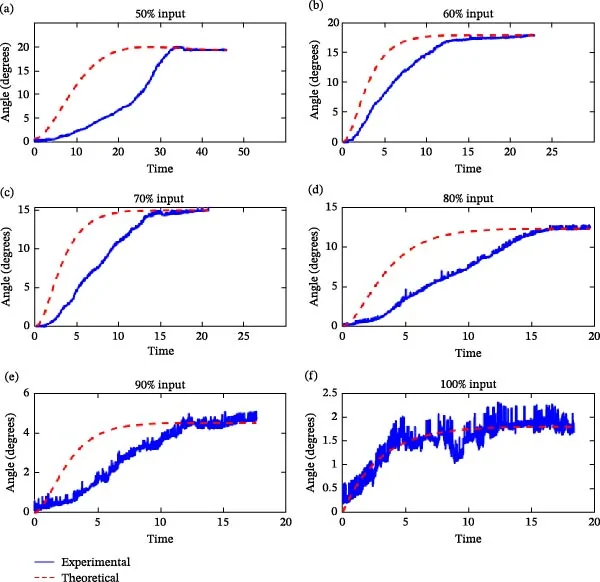
Comparison of experimental and simulated responses of the system adjusted to second‐order behaviors. (a) Experimental vs. theoretical response at 50% input. (b) Experimental vs. theoretical response at 60% input. (c) Experimental vs. theoretical response at 70% input. (d) Experimental vs. theoretical response at 80% input. (e) Experimental vs. theoretical response at 90% input. (f) Experimental vs. theoretical response at 100% input.

### 3.6. Model Refinement and Adjustment

The presence of a dead zone (0%–40%), nonlinear gain variation, and distinct damping characteristics across steps indicates that the system does not behave as a single linear time‐invariant (LTI) system across the full operating range.

For this reason, a piecewise nonparametric identification strategy was adopted. Each step interval was treated as a local linear approximation, resulting in a set of models that collectively describe the system behavior over the operational range (40%–100%). This approach improves modeling accuracy while preserving analytical simplicity for controller design.

Although the second‐order models significantly improved the fitting accuracy, additional refinements were introduced to enhance settling time prediction and better align the onset of the simulated response with experimental data. Small delays were incorporated to account for the pneumatic actuation lag. The refined transfer functions are presented in Table 9.

**Table 9:** Adjusted second‐order non‐parametric model for each input step.

Step	Transfer function
40–50	$\left(e^{-16s}\right)\frac{0.05431}{s^{2}+0.250.02905s+}$
50–60	$\left(e^{-s}\right)\frac{0.702}{s^{2}+1.950.3922s+}$
60–70	$\left(e^{-2s}\right)\frac{0.5326}{s^{2}+2.10.3527s+}$
70–80	$\left(e^{-2.3s}\right)\frac{0.371}{s^{2}+1.90.3017s+}$
80–90	$\left(e^{-1.8s}\right)\frac{0.2172}{s^{2}+1.90.4826s+}$
90–100	$\frac{0.18}{2.991s+}$

The improved fit between the experimental and adjusted model responses is shown in Figure 19.

**Figure 19:**
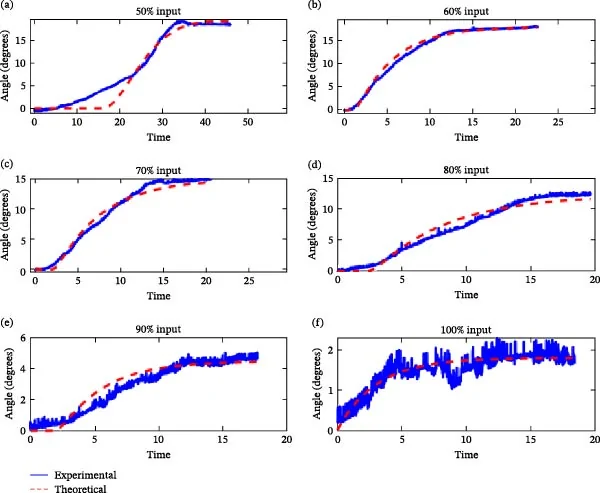
Comparison of the experimental response and the response simulated using the adjusted and modified equations to achieve a better fit. (a) Experimental vs. theoretical response at 50% input. (b) Experimental vs. theoretical response at 60% input. (c) Experimental vs. theoretical response at 70% input. (d) Experimental vs. theoretical response at 80% input. (e) Experimental vs. theoretical response at 90% input. (f) Experimental vs. theoretical response at 100% input.

### 3.7. Open‐Loop Control

To validate the accuracy of the identified models, a comparison was conducted between the simulated open‐loop response and the corresponding experimental measurements. The simulation reproduces the system response to increasing step inputs applied to the valve opening, allowing direct evaluation of the identified transfer functions under the same excitation conditions used during experimentation. The results of this comparison are presented in Figure 20.

**Figure 20:**
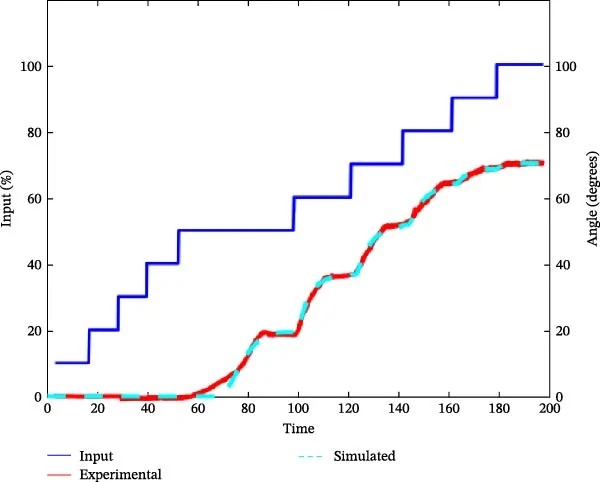
Simulated and experimental system responses.

The dynamic behavior obtained from simulation exhibits strong qualitative agreement with the experimentally observed response and with the previously derived non‐parametric models. As shown in Figure 19, the steady‐state values predicted by the identified transfer functions closely match the real system behavior across the tested operating range. Although minor discrepancies are observed during the transient phase, particularly in the rise time and damping characteristics, these differences do not significantly affect the functional performance of the rehabilitation device. Based on the graphical comparison between the simulated (red) and experimental (cyan) responses, the mean squared error (MSE) is ~2.0 (deg^2^), corresponding to a RMSE of about 1.4°. This relatively low error further supports the adequacy of the identified model in representing the system behavior.

Overall, the open‐loop validation confirms that the identified models provide an accurate representation of the system dynamics, particularly under steady‐state conditions, and are therefore suitable for subsequent control design and implementation.

## 4. Conclusion and Future Work

This work demonstrates that soft robotics constitutes a viable and effective alternative for upper‐limb rehabilitation devices. Through the evaluation of different materials and fabrication techniques, it was shown that the primary objective of elbow rehabilitation, namely, controlled flexion and extension within physiological limits, can be achieved at a performance level comparable to that of conventional commercial systems. Importantly, the use of compliant materials enhances intrinsic safety by eliminating rigid metallic components, thereby reducing the risk of injury associated with mechanical failure, inadequate maintenance, or improper use of traditional rigid rehabilitators.

Soft pneumatic actuators, specifically pouch motors, were investigated for their capability to generate smooth and natural elbow movements across the physiological range of motion. Their versatility enables actuation using different working fluids and supports various manufacturing approaches. In this study, sewing‐based fabrication demonstrated superior structural performance compared to thermal sealing, improving the resistance to internal pressure and mechanical loading. The stitched PVC‐based actuators exhibited reliable behavior under compression and sustained pressure without rupture. Although this manufacturing method increases production time and requires operator expertise, it significantly enhances the durability and operational robustness.

From a modeling perspective, the non‐parametric identification approach allowed systematic derivation of transfer functions representing the device dynamics across distinct operating regions. The transition from first‐order approximation to second‐order modeling was justified by the observed nonlinear and input‐dependent characteristics of the system. The final identified models showed good agreement with experimental responses, characterized by low steady‐state discrepancies and acceptable transient deviations. Open‐loop validation confirmed that the identified models provide an adequate representation of the system dynamics at this stage of development, although residual steady‐state error remains a limitation to be addressed.

Future work will focus on improving system performance through targeted enhancements, including upgrading the flow‐control valve motor to increase response speed, redesigning the valve assembly to match updated actuation requirements, and implementing closed‐loop control strategies to regulate nonlinear pneumatic behavior and eliminate steady‐state error. Furthermore, incorporating forearm supination and pronation mechanisms will enable more comprehensive rehabilitation capabilities. Following the implementation of closed‐loop control, extended operational testing and clinical validation in collaboration with rehabilitation centers were conducted to assess performance under real‐world conditions.

Overall, this research represents a meaningful advancement toward the development of affordable, safe, and adaptable soft robotic rehabilitation systems. By leveraging low‐cost materials and simplified mechanical architectures, the proposed prototype provides a promising alternative for deployment in developing countries and low‐resource settings, where access to conventional rehabilitation technologies remains limited. Its scalability and modular design support continued refinement and potential translation into practical clinical applications.

## Funding

This study was supported by the Universidad Iberoamericana Ciudad de México.

## Conflicts of Interest

The authors declare no conflicts of interest.

## Supporting Information

Additional supporting information can be found online in the Supporting Information section.

## Supporting information


**Supporting Information** Video S1: Demonstration of the soft robotic elbow rehabilitation device presented in this study. The video illustrates the integrated prototype, its main components, and its operation during the assisted elbow flexion–extension movement.

## Data Availability

The data that support the findings of this study are available from the corresponding author upon reasonable request.
